# Low muscle mass in lung cancer is associated with an inflammatory and immunosuppressive tumor microenvironment

**DOI:** 10.1186/s12967-023-03901-5

**Published:** 2023-02-11

**Authors:** Sarah Santiloni Cury, Diogo de Moraes, Jakeline Santos Oliveira, Paula Paccielli Freire, Patricia Pintor dos Reis, Miguel Luiz Batista, Érica Nishida Hasimoto, Robson Francisco Carvalho

**Affiliations:** 1 Department of Structural and Functional Biology, Institute of Biosciences, São Paulo State University (UNESP), Botucatu, São Paulo CEP: 18.618-689 Brazil; 2 Department of Biochemistry and Tissue Biology, University of Campinas, Rua Monteiro Lobato, 255, Campinas, São Paulo 13083-862 Brazil; 3 Department of Immunology, Institute of Biomedical Sciences, University of São Paulo, São Paulo, SP Brazil; 4 Department of Surgery and Orthopedics, Faculty of Medicine, São Paulo State University (UNESP), Botucatu, São Paulo 18618687 Brazil; 5 Department of Biochemistry, Boston University School of Medicine, Boston, USA

**Keywords:** Non-small cell lung cancer, Machine learning, Computed tomography, Transcriptomics, CD8+ T cells

## Abstract

**Background:**

Computed tomographies (CT) are useful for identifying muscle loss in non-small lung cancer (NSCLC) cachectic patients. However, we lack consensus on the best cutoff point for pectoralis muscle loss. We aimed to characterize NSCLC patients based on muscularity, clinical data, and the transcriptional profile from the tumor microenvironment to build a cachexia classification model.

**Methods:**

We used machine learning to generate a muscle loss prediction model, and the tumor's cellular and transcriptional profile was characterized in patients with low muscularity. First, we measured the pectoralis muscle area (PMA) of 211 treatment-naive NSCLC patients using CT available in The Cancer Imaging Archive. The cutoffs were established using machine learning algorithms (CART and Cutoff Finder) on PMA, clinical, and survival data. We evaluated the prediction model in a validation set (36 NSCLC). Tumor RNA-Seq (GSE103584) was used to profile the transcriptome and cellular composition based on digital cytometry.

**Results:**

CART demonstrated that a lower PMA was associated with a high risk of death (HR = 1.99). Cutoff Finder selected PMA cutoffs separating low-muscularity (LM) patients based on the risk of death (*P*-value = 0.003; discovery set). The cutoff presented 84% of success in classifying low muscle mass. The high risk of LM patients was also found in the validation set. Tumor RNA-Seq revealed 90 upregulated secretory genes in LM that potentially interact with muscle cell receptors. The LM upregulated genes enriched inflammatory biological processes. Digital cytometry revealed that LM patients presented high proportions of cytotoxic and exhausted CD8+ T cells.

**Conclusions:**

Our prediction model identified cutoffs that distinguished patients with lower PMA and survival with an inflammatory and immunosuppressive TME enriched with inflammatory factors and CD8+ T cells.

**Supplementary Information:**

The online version contains supplementary material available at 10.1186/s12967-023-03901-5.

## Background

Cachexia is a metabolic syndrome found in 50% of patients with lung cancer [1]. It is associated with the risk of death in non-small cell lung cancer (NSCLC) [2]—the most prevalent histological type [3]. Low muscle mass in cachectic patients with advanced NSCLC is associated with pain and poor quality of life [4]. Therefore, early detection of cachexia is of utmost importance in predicting NSCLC patient outcomes and guiding better treatment decisions.

Multiple screening tools are used to measure muscle loss [5]. One feasible approach is computed tomography (CT), which is routinely performed for cancer diagnosis and follow-up [6]. CT-based muscle quantification at the third lumbar vertebra (L3) level is commonly used to assess muscle mass in cancer patients [1]. However, CT scans of NSCLC patients usually do not include L3 [7]. Studies overcame this limitation by analyzing the pectoralis muscle area (PMA) [8, 9]. Our group demonstrated that PMA alone is a cachexia predictor in treatment-naive NSCLC patients [9]. PMA quantification in lung cancer patients is associated with clinical outcomes; however, each study used different calculations and cutoffs to select cachectic patients [8, 9]. Although informative, the best PMA cutoff that classifies cachectic patients based on CTs remains to be defined.

Molecular markers also contribute to the clinical classification of cachexia; however, none are yet approved for clinical use. The synergic expression of tumor-derived cachexia-inducing factors (CIFs) is correlated with the prevalence of cachexia in different tumor types [10]. For example, lung cancer cachexia is characterized by increased expression of CIFs that ultimately induce muscle wasting [11]. NSCLC patients with low PMA present increased expression of *IL8*, *IL6*, and other critical CIFs [9]. Cell types in the tumor microenvironment (TME) contribute distinctively by secreting cytokines or other CIFs that act on cell surface receptors of target tissues [12]. Cancer samples can be divided into clinically relevant immune subtypes depending on TME composition and gene expression profile [13]. Thus, computational deconvolution of the transcriptome from tumor bulk using digital cytometry and single-cell RNA-Seq (scRNA-Seq) can help infer the TME cellular proportions. TME analysis also has the potential to identify cells responsible for secreting CIF in cachectic patients.

The aim of this study was to build a machine learning (ML) model based on PMA and clinical data to identify NSCLC patients with low muscularity and poor prognosis. We additionally characterized the global secretome and TME immune fraction of these patients by utilizing tumor transcriptomes. Overall, the predictive model determined the best PMA cutoffs to select potentially cachectic patients. We demonstrated that these patients have a pro-inflammatory state characterized by high tumor gene expression of CIFs. Based on computational deconvolution of bulk transcriptomes, we also identified that patients with lower PMA and worse survival show a TME enriched with cytotoxic and exhausted CD8+ T cells.

## Methods

Software, tools, and databases are described in Additional file 1.

### NSCLC patients

CTs and clinical data were downloaded from The Cancer Imaging Archive (TCIA) database. The NSCLC-Radiogenomics collection contains 211 NSCLC patients [14, 15] (discovery set). CTs were taken at diagnosis, and patients were treated with surgery. We included 107 patients with primary tumors, presenting whole-body PET-CT before surgery, not subjected to chemo or radiotherapy, with arms raised during CT to avoid positioning bias, and overall survival data. An independent validation set with 36 primary NSCLC patients (all gave their informed consent prior to their inclusion in the study) from the Faculty of Medicine, São Paulo State University (UNESP)—Botucatu, Brazil (2012–2019) was used to confirm the prediction model (approved by the Faculty of Medicine Research Ethics Committee REB#45723921.0.0000.5411). Third-two patients from the validation set had survival data.

### CT analyses

We analyzed the PMA using a single axial slice of the CT as previously described [9]. We analyzed the total skeletal muscle cross-sectional area at the level of the L3 to classify patients with low muscle mass based on pre-established skeletal muscle indexes (SMI; men < 55 cm^2^/m^2^; women < 39 cm^2^/m^2^) [4]. This classification was compared with the one generated by our model. We generated PMA-SMI (cm^2^/height^2^) to test whether height normalization affects our prediction model.

### Cachexia classification model using machine learning

Predictive analysis based on a non-parametric risk prediction model was performed by the Classification And Regression Tree (CART) method using the regression analysis available in "rpart" using RStudio. We performed pruning to generate the tree with minimum × error (prune function; cp = 0.053). We used ten-fold cross-validation to assess the possibility of overfitting. We analyzed the age, tumor stage, PMA, and survival as variables. The imbalance between the two groups was addressed by a balanced resampling using random down-sampling. The Cutoff Finder package was independently applied to select the PMA cutoff that distinguishes patients' survival using R. Area Under the Curve-Receiver Operating Characteristics (AUC-ROC) analysis with a 95% confidence interval (CI) was used via the easyROC web tool to determine the diagnostic value of our PMA cutoffs in predicting low muscle mass. For this comparison, muscularity was defined using L3 SMI cutoffs. EasyROC was also used to test PMA-indexes in determining the LM and HM patients.

### Tumor transcriptomic analysis

The tumor RNA-sequencing data (Illumina HiSeq 2500) of 130 subjects from NSCLC Radiogenomics collection were publicly available at GEO datasets (GSE103584). Of the 107 included patients, 46 had tumor transcriptomic data. We compared count data from LM patients (N = 9) with HM patients (N = 37) to perform differential gene expression analysis using the default setting of the BioJupies platform. The differentially expressed genes (DEGs) were selected according to fold change (FC) >|1.5| and *P*-value < 0.05. DEGs encoding secreted proteins were filtered based on the Human Protein Atlas database using the majority decision-based method for secreted proteins list of 2943 genes (Additional file 3: Table S1). Principal component analysis (PCA) was performed using the ClustVis webtool.

### Enrichment and protein–protein interaction (PPI)

We used EnrichR to perform a functional enrichment analysis of DEGs. We selected the top 10 most enriched biological processes using gene ontology (GO) - 2018. PPI networks were conducted using the STRING tool. We considered experiments, databases, co-expression, neighborhood, and co-occurrence as active interaction sources. Only interactions with the highest confidence (interaction score > 0.9) were included, and disconnected nodes were omitted from the final network. A *P*-value < 0.01 was considered statistically significant. PPI network data was visualized and annotated using Cytoscape. The network was analyzed using the CytoNCA plugin, which calculated the node's betweenness centrality.

### Tumor-muscle crosstalk prediction

We used the consensus list of ligand-receptors generated by Ramilowski et al. [16] to test if the skeletal muscles express receptors for the tumor-derived factors. We validated the gene expression of each predicted muscle receptor in human primary muscle cells from the FANTOM5 project, 491 muscle tissues from the GTEx portal, muscle tissues from the BioGPS website, muscle tissues from GeneAtlas U133A, and the gcrma human dataset. The alluvial diagram connecting the ligands to their receptors was generated using the SankeyMATIC tool.

### Expression profile of secretome genes in malignant cells

To evaluate the abundance of DEGs found in the tumor of LM in malignant lung cells, we used transcriptomic data from the Cancer Cell Line Encyclopedia (CCLE). We selected eight cell lines with higher correlation with tumor tissues using the "TCGA-110CL Cell Line Panel". Considering that lung tumor organoids recapitulate the histology, gene expression, and genomic profile of the original tumor [17], we included lung tumor-derived organoids transcriptomic data available in the HCMI (Human Cancer Model Initiative) catalog (duplicates of HCM-CSHL-0058-C34). The selected cell lines and organoids are described in Additional file 3: Table S2.

### Digital cytometry and scRNA-Seq

To evaluate whether immune infiltrating cells express the DEGs found in the LM, we performed digital cytometry analysis using the CIBERSORTx tool to impute the immune cell fractions of 22 cell types (LM22 matrix signature) from the discovery bulk RNA-Seq data. We applied the default settings and batch-correction. We also performed a gene expression imputation analysis to verify whether the over-expressed genes in LM patients are expressed in the six major leukocyte subsets (B cells, CD4+ T cells, CD8+ T cells, NKT cells, NK cells, and monocytes) of patients with NSCLC derived from scRNA-Seq data [18]. The gene expression profile (GEP) was estimated by CIBERSORTx group mode analysis. In addition, the specific state of T cells (naive, cytotoxic, and exhausted) was evaluated using Immune Cell Abundance Identifier (ImmuCellAI).

### Ligand–receptor interactions from gene expression

We used the computational framework CellPhoneDB to predict cell–cell communication and better understand the interplay of tumor immune cells using its repository of curated ligand–receptor interactions for scRNA-Seq. We used the default setting to select the statistically relevant interaction (*P*-value < 0.05) between the NSCLC T cells using the expression profile enriched in LM patients. This analysis was performed by re-analyzing the publicly available scRNA-Seq data from Guo et al. [19] (GSE99254).

### Statistical analyses

Statistical analysis not previously reported was performed using GraphPad Prism® (GraphPad Software, v5.0, 2008 USA). The Mann–Whitney U-test was applied to compare the body composition variables between LM and HM patients (applied for non-parametric data). Chi-square analysis was used for categorical variables. Overall survival analysis was performed using the Kaplan–Meier method, and the log-rank test was used to compare the curves. Differences with a *P*-value < 0.05 were considered significant.

## Results

### Predictive models identify the best PMA cutoff that classifies low muscle mass

We included 107 patients with NSCLC with a mean age of 68.7 years old (42 to 87). Sixty-eight were male, and 39 were female. Sex differences were considered when analyzing the PMA. We also identified differences according to the age range, especially in women; the older ones (> 65 years old) presented lower PMA than younger ones (≤ 65 years) (Additional file 2: Fig. S1A). PMA did not differ between young and older men (Additional file 2: Fig. S1A).

**Fig. 1:**
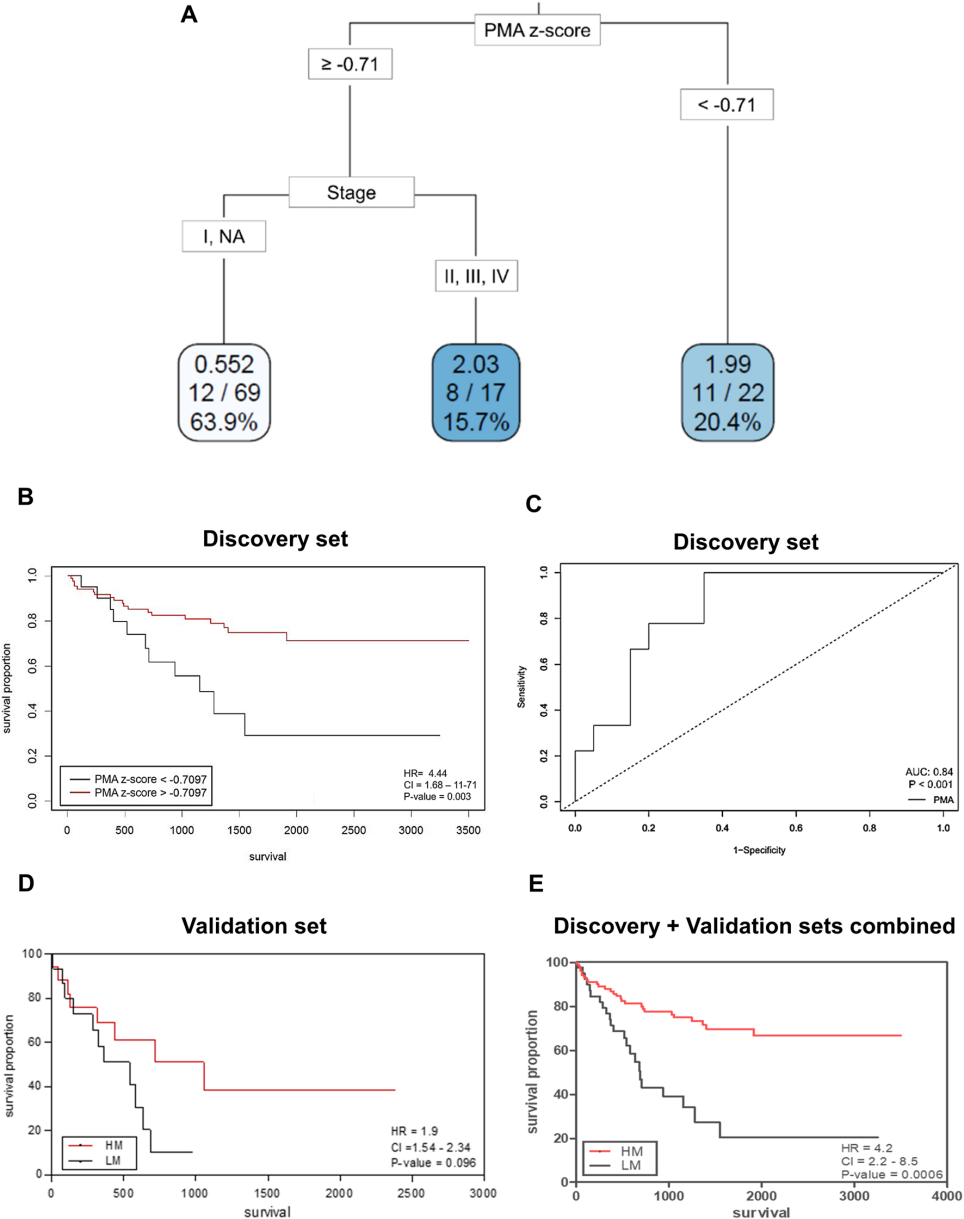
Cachexia classification models. **A** Decision tree generated using Classification And Regression Trees (CART). The bottom boxes indicate hazard ratios, the number of patients at risk in each leaf, and the percentage of patients in each leaf. This analysis was performed using the discovery set samples. **B** Kaplan–Meier survival curve generated by Cutoff Finder tool (red-curve: PMA > 0.71 and low-risk group; black-curve: PMA < 0.71 and high-risk group) using samples from the discovery set. **C** AUC-ROC curve demonstrating the specificity and sensitivity of PMA cutoffs in indicating low muscle mass (determined by third lumbar skeletal muscle index cutoffs [4]) in the discovery set. **D** Kaplan–Meier survival curve comparing LM (low-muscularity patients) and HM (high-muscularity patients) using samples from the validation set. Red-curve: PMA > 0.71 and low-risk group; Black-curve: PMA < 0.71 and high-risk group The resulting *P*-values for the log-rank test are shown. **E** Kaplan–Meier survival curve comparing low- and high-muscularity (LM and HM, respectively) patients by combining the discovery and validation datasets. Red-curve: PMA > 0.71 and low-risk group; Black-curve: PMA < 0.71 and high-risk group. The resulting P-values for the log-rank test are shown. HR: hazard ratio; CI: 95% confidence interval

We normalized PMA with the z-score considering sex and age differences to make the groups comparable (Additional file 2: Fig. S1B). The CART analysis showed that patients with lower PMA are associated with a high risk of death followed by tumor stage (Fig. 1A). Using the Cutoff Finder, the same muscularity cutoff separated patients with poor survival and low PMA (low-muscularity patients, LM; n = 22) from patients with better survival and high PMA (high-muscularity patients, HM; n = 85) (Fig. 1B). Down-sampling for balanced comparisons using CART demonstrated models with similar cutoffs able to separate high-risk patients (Additional file 2: Fig. S1C). Using the L3 SMI as a muscularity parameter, we found that PMA cutoffs classify patients with low muscle mass with an AUC value of 0.84 (95% CI: 0.7–0.98; P < 0.001; Fig. 1C). Considering the relevance of height normalization, we also generated PMA-SMI. The LM group also presented a significant decrease in PMA-SMI (Table 1), and these values were consistent with the PMA classification for men and women (Additional file 2: Fig. S1D). Therefore, we conclude that both PMA and PMA height-normalized are good predictors.

**Table 1:** Clinical findings and skeletal muscle parameters of patients with NSCLC (non-small cell lung cancer) with low- and high-muscularity defined by pectoralis muscle cross-sectional area assessed using computed tomography and classification predictive model

	Discovery set			Validation set			Statistical test
	LM (PMA < -0.71) N = 22	HM (PMA > -0.71) N = 85	p-value	LM N = 19	HM N = 17	p-value	
Age	68.5 (9.5)	69 (11)	0.56	63 (4)	67 (6)	0.82	U test
PMA (cm^2^)	30.3 (9.3)	37.9 (16.4)	< 0.0001	23.9 (8.4)	32.1 (13.3)	0.036	U test
BMI (kg/m^2^)	21.8 (6.3)	26.5 (5.5)	0.0005	–	–	–	U test
SMI (cm^2^/m^2^)	43.3 (6.8)	50.7 (10)	0.020	–	–	–	U test
PMA-SMI (cm^2^/m^2^)	9.8 (2.4)	13.3 (2.91)	< 0.0001	–	–	–	
Gender (%)							
Male	72.7	61.2	0.32	68.4	35.3	0.047	Chi-square
Female	27.3	38.8		31.6	64.7		
Ethnicity (%)							
Caucasian	72.7	55.3	0.27	–	–	–	Chi-square
Asian	4.5	16.5		–	–		
African American	4.5	1.2		–	–		
Hispanic/Latino	0	3.5		–	–		
Native Hawaiian	4.5	1.2		–	–		
NA	13.6	22.3		–	–		
Smoking history (%)							
Current	36.4	10.7	0.014	100	70.6	0.011	Chi-square
Former	50	66.7		–	–		
Nonsmoker	13.6	22.6		0	29.4		
Histology (%)							
Adenocarcinoma	72.7	85.7	0.15	63.1	41.2	0.18	Chi-square
Squamous cell carcinoma	27.3	14.3		36.8	58.8		
Stage (%)							
I–II	94.7	93.6	0.86	73.7	58.8	0.34	Chi-square
III–IV	5.3	6.4		26.3	41.2		
Grade (%)							
G1	21	37.9	0.008	23.5	42.8	0.25	Chi-square
G2	47.4	56.1		70.6	51.2		
G3	31.6	6		5.9	–		
Lymphovascular invasion (%)							
Yes	11.8	5	0.32				Chi-square
No	88.2	95		73.7	68.7	0.74	
Pleural invasion (%)				26.3	31.3		
Yes	31.6	12.3	0.047	58.8	40	0.29	Chi-square
No	68.4	87.7		41.2	60		
Recurrence (%)							
Yes	36.4	14.3	0.018	42.1	23.5	0.24	Chi-square
No	63.6	85.7		57.8	76.5		
EGFR mutation status (%)							
Wildtype	82.3	71	0.3441	–	–	–	Chi-square
Mutant	17.7	29		–	–		
KRAS mutation status (%)							
Wildtype	68.7	83.1	0.1973	–	–	–	Chi-square
Mutant	31.3	16.9		–	–		

N: number of patients; PMA: Pectoralis Muscle Area; LM: low-muscularity patients; HM: high-muscularity patients; BMI: body mass index; SMI: skeletal muscle index (L3 muscles). The data represent the median (interquartile range, IQR) or the percentage

By transforming the z-scored PMA values into absolute values, we established the PMA cutoffs to select LM patients according to age and sex: young male = PMA < 34.4 cm^2^; old male = PMA < 34.3 cm^2^; young female = PMA < 25.4 cm^2^; and old female = PMA < 18.6 cm^2^. LM patients had lower body mass index (BMI), SMI, and PMA-SMI. We observed increased smokers' frequency, high tumor grade, pleural invasion, and tumor recurrence in the LM patients (Table 1). An independent group of LM patients with NSCLC analyzed according to the PMA cutoffs generated during the discovery analysis confirmed the associations with poor prognosis (worse survival, recurrence, and smoking habits) (Table 1 and Fig. 1D). By combining the survival data of patients from the discovery and validation sets, we found a more significant log-rank P-value < 0.001 (Fig. 1E). Although not statistically significant, more patients with LM (31.3%) had *KRAS* mutations compared to HM (16.9%).

### Tumors from patients with LM present a pro-inflammatory profile

**Fig. 2:**
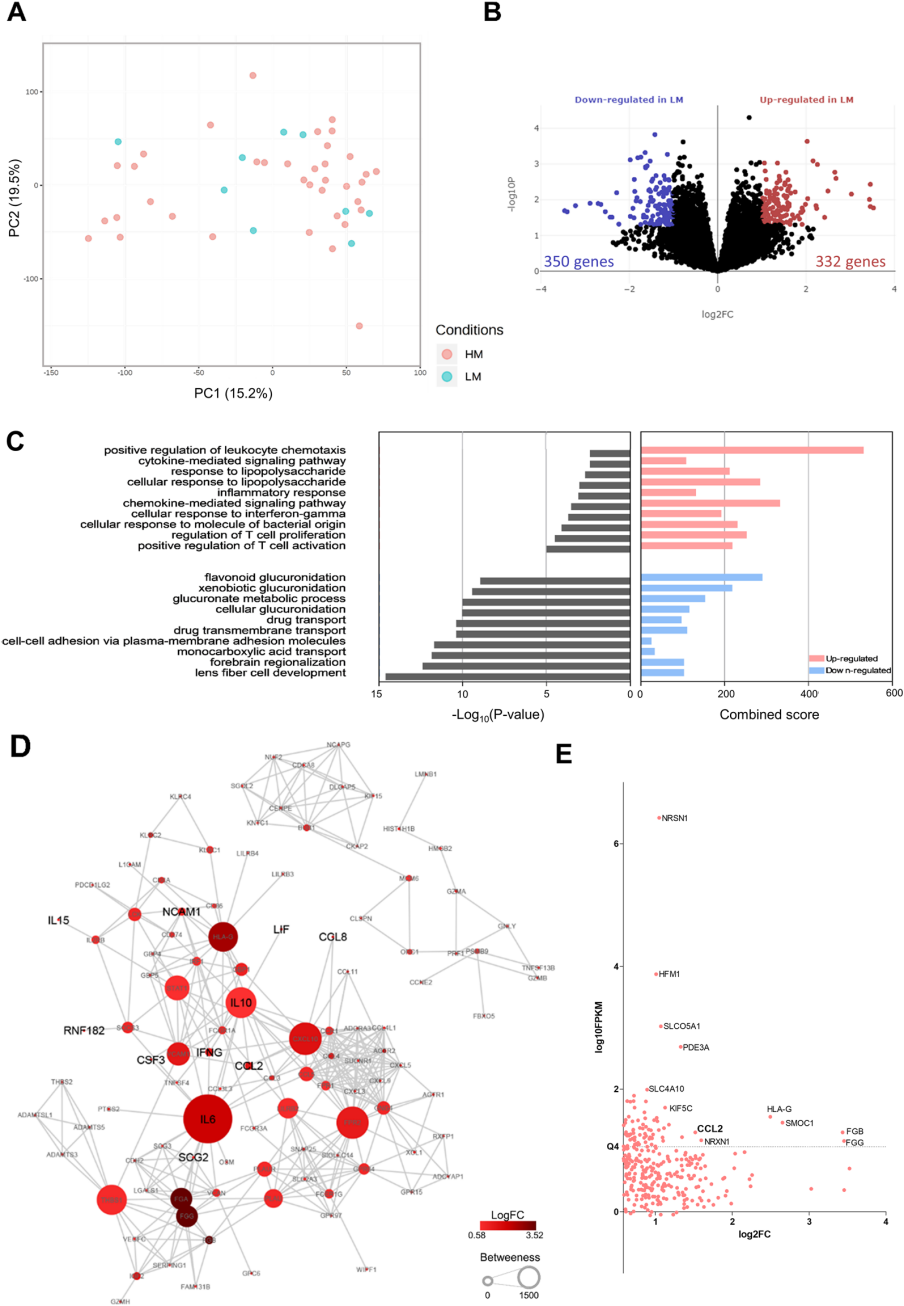
Tumors from low-muscularity (LM) patients present a pro-inflammatory profile enriched with cachexia-inducing factors and mediators. **A** Principal component analysis (PCA) demonstrating similar expression profiles between high-muscularity (HM) and LM patients. **B** Volcano-plot demonstrating differentially expressed genes in LM (fold change > 1.5 and *P*-value < 0.05). **C** Enrichment analysis of top 10 terms related to biological processes from 332 up (red) and 350 downregulated (blue) genes in LM. Bar graphs represent ranked − log10 *P*-values (Fisher exact test), from the least to the most significant according to EnrichR analysis. **D** Protein–protein interaction networks of the ninety upregulated genes predicted to encode secreted proteins in LM patients. The larger the circles, the higher the betweenness centrality value of the node. Red intensities represent the gene expression fold change (logFC). Larger letter sizes indicate previously described cachexia-inducing factors in pan-cancer [10] and lung cancer [9]. Gray lines highlight the interactions. Interactions were visualized using Cytoscape v3.7.2. **E** Scatterplot comparing abundance (log10 FPKMs, y-axis) and degree of expression (log2-fold change, x-axis) of upregulated genes in LM. FPKM: Fragments Per Kilobase Million

We included nine LM patients and 37 HM patients with RNA-Seq data availability for the expression analysis and observed a similar expression profile using the PCA (Fig. 2A). These similarities resulted in considering the *P*-values < 0.05 to select DEGs for further analysis. We took into consideration the biological context of these DEGs to avoid misinterpretation due to false positives [20–22]. We identified 682 DEGs (332 were up and 350 were downregulated) in LM (Fig. 2B and Additional file 3: Table S3). Enrichment analysis of upregulated genes demonstrated leukocyte chemotaxis, cytokine/chemokine activity, and T-cell activation as the most enriched terms. The downregulated genes enriched terms associated with drug transport, cellular adhesion, and glucuronidation (Fig. 2C). We found 90 and 41 up and downregulated secretome genes in LM (Additional file 3: Table S3). We failed to identify significant interactions among the downregulated secretory genes in the PPI. Therefore, we focused on upregulated secretome genes that included several known cachexia mediators described in pan-cancer [10] and NSCLC [9] (Additional file 3: Table S4), such as *IL6, IFNG, LIF*, *CCL2,* and *CSF3* (Fig. 2D). IL6 presented a central position in the network. *CCL2* (MCP‐1) was the CIF with the highest abundance in LM tumors (Fig. 2E).

### Tumor-derived factors are predicted to interact with skeletal muscle

**Fig. 3:**
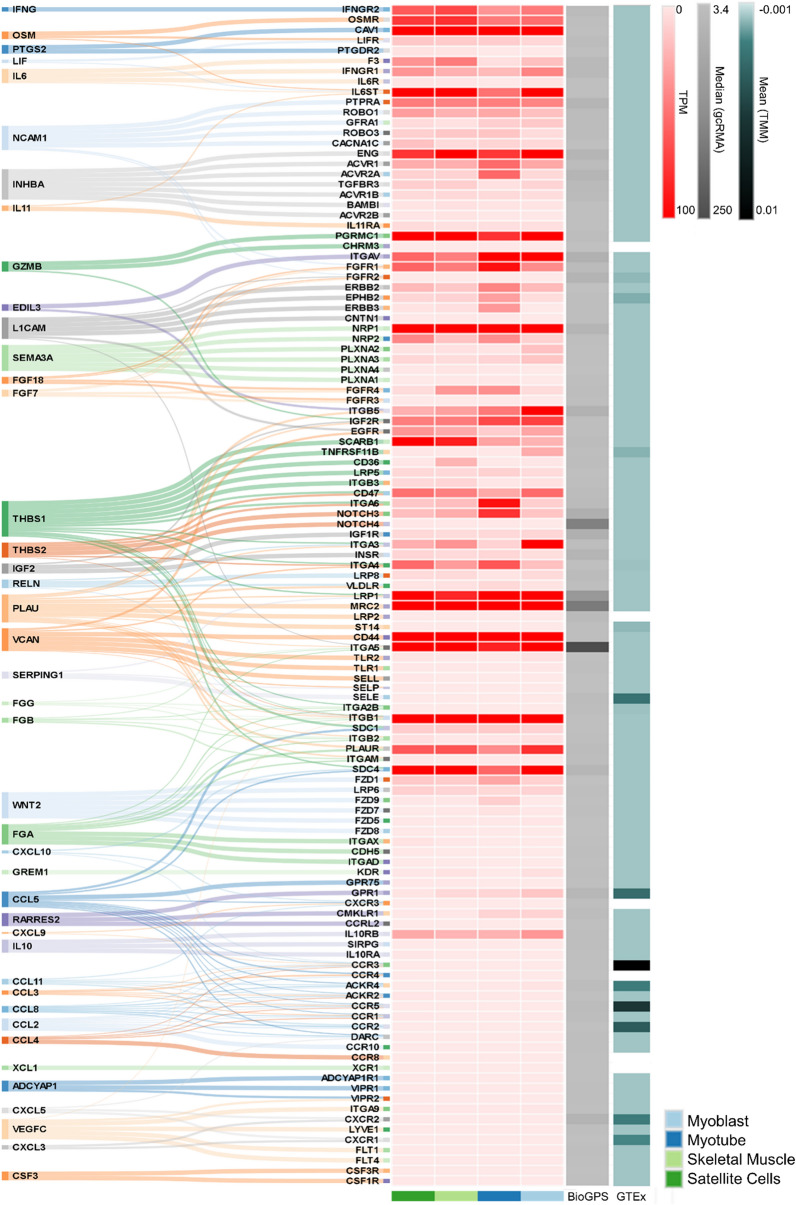
Tumor-derived factors and skeletal muscle connectome. Left: alluvial diagram connecting the ligands from the upregulated genes in LM into its matched skeletal muscle receptors. Right: Heatmap of the expression levels of predicted receptors in human primary cell lineages, previously described by Ramilowski et al. [16] (red: Transcripts Per Million—TPM), four skeletal muscle tissues from BioGPS (gray: median gcRMA), and 491 skeletal muscle samples from the Genotype-Tissue Expression (GTEx) project (green: Trimmed Mean of M-values—TMM)

To verify how the secretome profile from tumors of LM is potentially altering the skeletal muscle morphology, we constructed a tumor-muscle connectome by identifying the gene expression of the ligands produced by the tumors and their corresponding receptors in skeletal muscle cells and tissues (Fig. 3). We observed that several ligands have receptors expressed in abundance both in skeletal muscle cells and tissues. Interestingly, *LIF*, *IFNG,* and *IL6* were the cytokines presenting receptors with high expression in muscle cells compared to other cytokines, such as CCLs and CXCLs, in which receptor expression is absent in muscle cell lines. Many receptors for CCLs and CXCLs are expressed in skeletal tissues, as demonstrated in the GTEx and BioGPS data, suggesting that these cytokines target muscle tissue cells other than myoblasts, myotubes, and satellite cells. This analysis ratifies that tumor-released factors may target skeletal muscle via specific receptors.

### Malignant cells express low levels of inflammatory genes

**Fig. 4:**
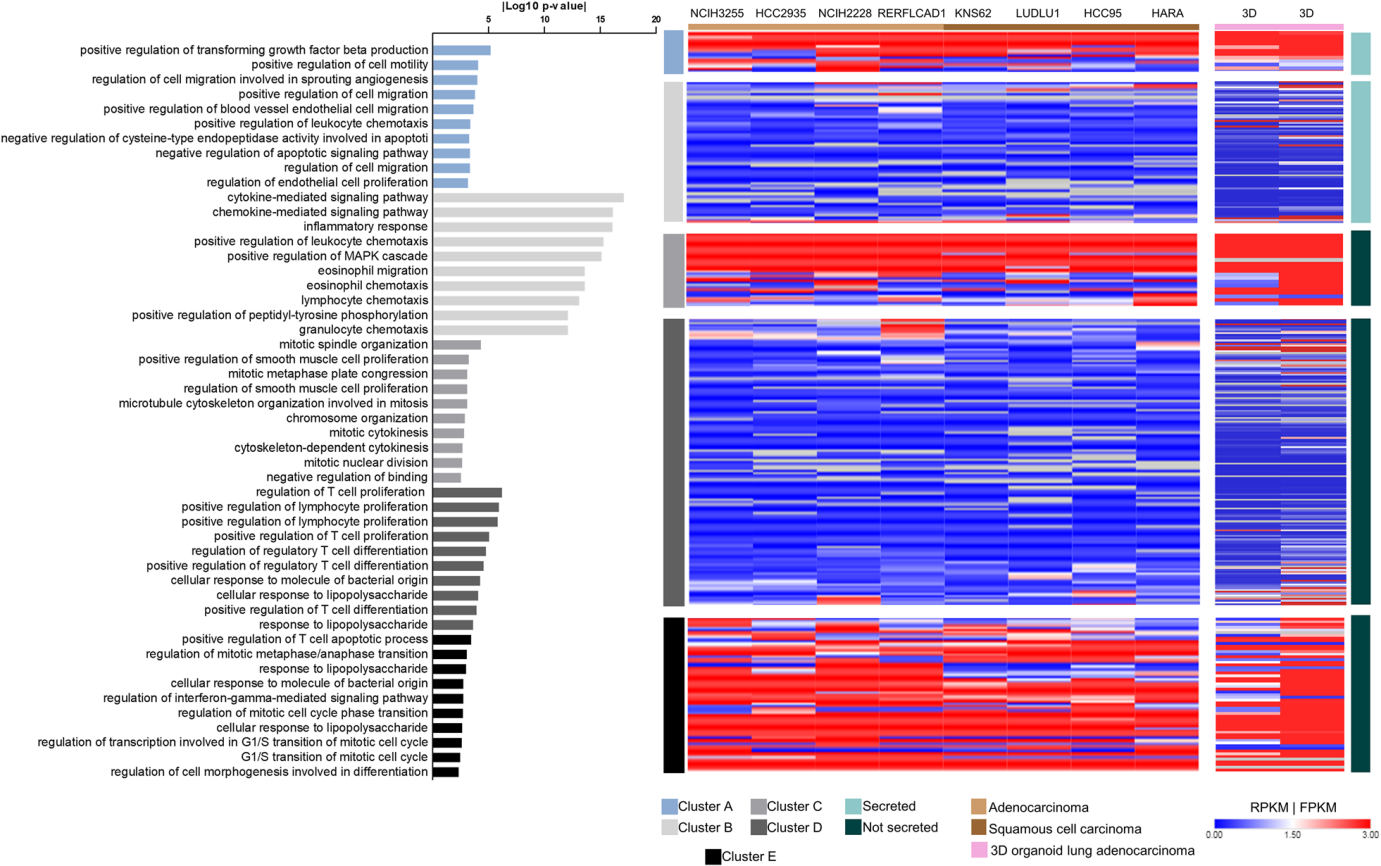
Tumor secretome derived from non-malignant NSCLC cells. Left: Enrichment analysis of the top 10 terms related to biological processes of each cluster according to EnrichR analysis. Right: Heatmap of gene abundance from cell lines (Reads Per Kilobase Million—RPKM) and lung adenocarcinoma derived-organoid duplicates (Fragment Per Kilobase Million—FPKM) in five clusters generated using k-means clustering analysis of secreted and non-secreted encoding genes. Global expression profiles of eight CCLE cell lines (Cancer Cell Line Encyclopedia) derived from adenocarcinoma (N = 4) and squamous cell carcinomas (N = 4) were evaluated. Global gene expression profiles of 3D organoids were collected from a single patient available in the HCMI catalog (Human Cancer Model Initiative)

Once we identified a cachexia-like expression profile in LM tumors, we next investigated the tumor cell types responsible for producing CIFs. First, to evaluate whether the malignant cells express the 332 upregulated genes found in the LM, we investigated the abundance of these DEGs using the expression profile of NSCLC cells (lines and organoids). We applied k-means clusterization and identified five clusters. We detected two clusters in secretome genes: cluster A represents abundant secretory genes in malignant cells, and cluster B represents low-level of secretory genes in malignant cells. The genes in cluster A enriched angiogenesis and leukocyte chemotaxis (*THBS1* and *VEGFC*), and the genes present in cluster B are associated with inflammation. Among the genes not predicted to encode secreted proteins, we identified three clusters: clusters C and E, representing genes abundant in malignant cells associated with cell proliferation and mitosis, and cluster D, containing genes expressed at low levels in malignant cells and associated with T cell biology. Noteworthy, cluster E enriched the term "positive regulation of T cell apoptotic process" associated with *IDO1* and *CD274* (PDL1) (Fig. 4).

### LM patients present high proportions of CD8+ T cells

**Fig. 5:**
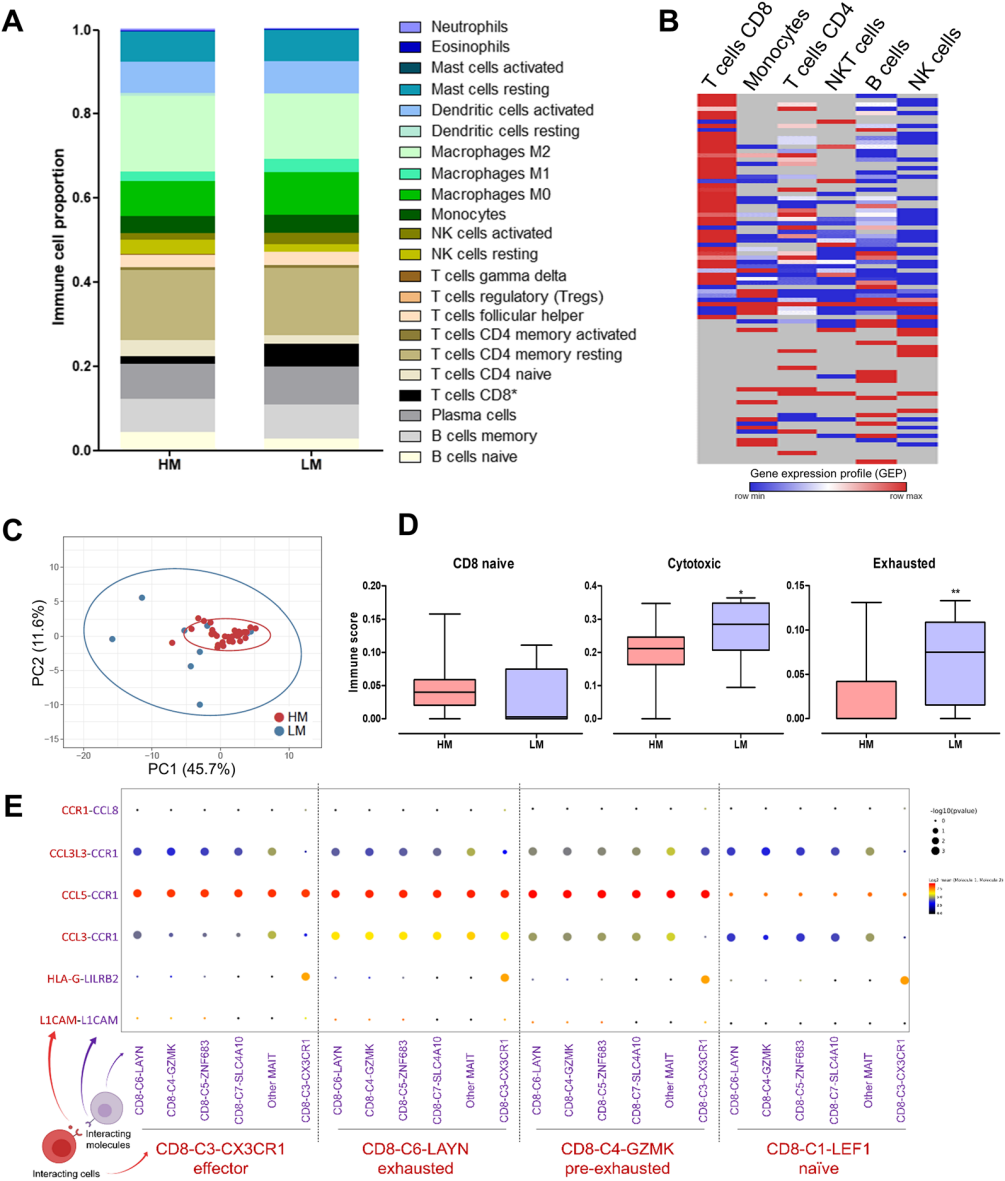
Digital cytometry based on the gene expression profile revealed that LM patients presented high proportions of CD8+ T cells. **A** Bar graph representing 22 immune cell scores in patients with LM and HM calculated using CIBERSORTx. *P-value = 0.037 (Mann–Whitney). **B** Gene expression profiles (GEP) of secretome genes found upregulated in tumor tissue of LM patients analyzed in single-cell RNA-Seq in peripheral blood mononuclear cells (PBMCs) of NSCLC patients. GEP represents the estimated expression values (log2) calculated by CIBERSORTx using the group mode function. **C** Principal component analysis (PCA) demonstrating HM and LM clusters generated using the CD8+ T cells enriched expression profile. **D** Box-plots of CD8 naive, cytotoxic, and exhausted cell scores between LM and HM patients determined using ImmuCellAI. *P-value < 0.05. **E** Cell–cell communications between T cell subsets from NSCLC using the upregulated genes list enriched in LM patients (single-cell data of NSCLC T cell population from GSE99254). *P*-values are indicated by circle size. The average expression level of interacting molecule 1 in CD8 subset red and interacting molecule 2 in CD8 subset purple is indicated by color. Analysis was performed using CellPhoneDB. LM: low-muscularity; HM: high-muscularity

Since we observed that many inflammation-associated genes are expressed at low levels by the malignant cells, we used CIBERSORTx to predict immune cells that contribute to the expression of these genes. LM patients presented high proportions of CD8+ T cells (Fig. 5A). To understand the molecular profile of CD8+ T cells in the TME, we used an independent dataset of NSCLC scRNA-Seq to analyze the expression profile of the 90 secretome genes identified in LM patients. We found CD8+ T cells expressing several of these genes in higher proportions than other immune cells (Fig. 5B), suggesting that CD8+ T cells may express secretome genes.

When plotting PCA using the expression profile enriched by CD8+ T cells, we better separated HM patients from LM patients (Fig. 5C). Cytotoxic and exhausted T cells were prevalent in LM compared to HM (Fig. 5D). Investigating the 332 upregulated genes in LM in another scRNA-Seq data from the T cell population of NSCLC using CellphoneDB, we predicted ligands (CCL3L3, CCL5, CCL3, and HLA-G) and receptors (CCR1 and LILRB2) communications among effectors, exhausted, pre-exhausted, and naive CD8+ T cells (Fig. 5E). We observed that exhausted and pre-exhausted CD8+ T cells presented increased interactions, specifically via CCR1 (exhausted)—CCL3L3 (pre-exhausted) and CCL3 (exhausted)—CCR1 (pre-exhausted) (Additional file 2: Figs. S2A and 5E). Moreover, genes expressed in exhausted tumor-infiltrating CD8+ T cells [19] were upregulated in LM patients, such as *GZMB, VCAM1, IFNG, CCL3, FASLG, MYO7A, STMN1, SAMSN1*, and *ITGAE* (Additional file 3: Table S5). CD8 and exhaustion scores are negatively correlated with PMA (Additional file 2: Fig. S2B).

## Discussion

We showed that a lower PMA evaluated in CTs used for NSCLC diagnosis before tumor resection predicts worse outcomes in patients with NSCLC. We also proposed PMA cutoffs for women and men at young and older ages based on ML analysis. Tumor transcriptome demonstrated that LM patients present a cachexia-like profile highly pro-inflammatory and express CIFs. Digital cytometry based on transcriptomic data revealed that TME is enriched with a suppressive immune microenvironment, mainly composed of CD8+ T cells. The prediction of ligand-receptor revealed candidates that may ultimately lead to muscle wasting.

Cancer cachexia classification remains an important clinical challenge. The milestones for the classification of cachexia are weight loss > 5% over the past 6 months or BMI < 20 and any degree of weight loss > 2% or lumbar SMI determined by CT imaging (men < 55 cm^2^/m^2^; women < 39 cm^2^/m^2^) [4]. However, CTs are often taken in the thoracic region of lung cancer patients [7]. To overcome this limitation, we constructed a cachexia classification model using ML to evaluate clinical parameters routinely available for NSCLC patients. We found that lower PMA can predict worse survival. The PMA (z-scored) cutoff identified in CART was also validated by independent analysis. These results demonstrated that PMA cutoffs are strong predictors of the poor prognosis of NSCLC patients with low muscle mass. Despite not demonstrating an association with worse overall survival, our research group previously demonstrated PMA cutoffs as a potential cachexia predictor in NSCLC patients[9]. The cutoffs found in this study were PMA < 32.2 cm^2^ and < 21 cm^2^ for men and women, respectively, similar to those found herein using robust ML. CART was recently used to predict cachexia risk levels in incurable cancer patients with more than 88% of precision and sensitivity [23]. These results reinforce the importance of using ML approaches applied to clinical features of cancer patients to classify cachexia.

We found a high frequency of smokers in the LM group, which corroborates the literature that indicates an increase in muscle loss in tobacco smokers [24] and confirms the significant role of nicotine in altering the tumor microenvironment and prognosis, as demonstrated for cachectic patients with pancreatic cancer [25]. A high risk of tumor recurrence was found in LM patients and agreed with other cachexia studies [26]. *KRA*S mutation was frequently found in our LM patients, and evidence suggests *KRAS*-mutated tumors are commonly associated with lung cancer cachexia [27]. Since adenocarcinomas—the tumor with the highest prevalence of the *KRAS* mutation [28]—occur at similar rates in LM (72.7%) and HM (85.7%) groups, we confirmed that *KRAS* mutations were associated with muscularity rather than tumor histopathology. Thus, studies investigating differences in the cachexia phenotype of preclinical models inoculated with KRAS-mutated NSCLC cell lines may help to understand the impact of *KRAS* mutation in the syndrome. These essential features can help guide cancer cachexia definition towards precision medicine in the future.

Our cachexia classification was validated by the increase of CIFs genes in the tumor of LM patients, such as *LIF*, *IL6*, *IFNG*, and *CCL2* [10]. We also identified increased expression of genes described in NSCLC patients with lower PMA, such as *NCAM1*, *SCG2*, *CSF3*, and *CCL8* [9]. To understand how the secretome influences skeletal muscle phenotype and function, we characterized the landscape of cell–cell interaction between tumor and skeletal muscle cells using transcriptomic data from both tissues. This approach has a higher resolution than proteomics and provides insights into cell and tissue communication [29]. We found receptors for CIFs expressed at high levels in skeletal muscle cells, such as IFNG receptor (IFNGR2) and LIF receptors (LIFR and IL6ST), which can directly trigger atrophy [1]. The gp130 family members, IL-11, IL-6, OSM, and LIF (upregulated in the LM) potentially impact skeletal muscle wasting by inducing the STAT3 signaling pathway in the preclinical model of cancer cachexia [30]. These results confirm the importance of these tumor-derived factors in NSCLC cachexia. Previous studies have individually inhibited the classical CIFs described herein [31]. Although preclinical studies inhibiting CIFs have shown some efficiency, these factors have not provided the same benefit in clinical trials [31]. Our tumor-muscle interactome revealed a combinatorial action of secreted mediators from cells within the TME that directly acts on muscle wasting. More studies are needed to describe the causality of these ligand–receptors interactions and their combinatorial action in inducing muscle atrophy in NSCLC cachexia.

To investigate the tumor cells responsible for CIFs secretion, we further evaluated the expression profile of neoplastic lung cells. We found that inflammation-related genes are lowly expressed, while genes associated with vessel permeability and T-cell motility, recruitment, and activation are increased [32]. We identified enrichment in genes associated with T cell apoptosis, specifically the genes *CD274* (encoding PDL1) and *IDO1*. The processes involved in tumor immune evasion through IFN-γ (enriched in LM) have been reviewed [33]. Anti-tumor cells secrete IFN-γ that induces genomic instability and/or immune evasive gene expression signature in malignant cells (including *PDL1* and *IDO1*) [33]. PDL1 is the ligand of the inhibitory receptor PD-1, responsible for CD8 dysfunction in NSCLC [34]. IDO1 produces local depletion of amino acid tryptophan, contributing to the immunosuppressive microenvironment [33]. These results corroborate our digital cytometry that demonstrates an increase in CD8+ T cells in TME of LM patients enriched with exhausted cells but also enriched with cytotoxic T cells.

Although cytotoxic CD8+ T cells infiltrated in TME are frequently associated with intense anticancer activity [35], the CD8+ T cells' signal in tumor tissues is not always related to better outcomes. Using TCGA data, six immune subtypes were identified. Subtype 2 (C2) is characterized by IFN-γ predominance, the highest content of M1 macrophages and CD8+ T cells. Cancer patients presenting subtype C2 showed less favorable survival despite having the highest lymphocyte infiltration [13]. The authors proposed that these tumors are more aggressive or escape immune recognition by pre-existing remodeling. However, our results raise the possibility that patients with C2 tumors have less favorable survival due to cachexia. In fact, CD8+ T cells induce cachexia and adipose tissue wasting in experimental models of chronic infection via type I IFN [36].

Considering the immunosuppressive profile of LM tumors and immunotherapy promises to restore T cell cytotoxicity and its anticancer properties, immunotherapy would probably benefit NSCLC cachectic patients. Cachectic patients with NSCLC are resistant to immunotherapy [37]; thus, restoring the cytotoxicity of CD8+ T cells may be crucial for treatment response. Indeed, clinical trials testing an agent to reverse cachexia combined with an immune checkpoint inhibitor to treat advanced lung cancer patients is relevant since the treatment with ghrelin analog affects T cell development and proliferation [38]. Therefore, more studies are needed to verify whether immunotherapy combined with CIF-targeted drugs has the potential to reverse cachexia and increase the immunotherapy response.

## Strengths and limitations

This study is innovative and valuable for NSCLC cachexia. Here, we integrated imaging, clinical, and transcriptomic data to capture the complex interplay between tumor biology and host response associated with cachexia by applying bioinformatics and ML analyses. The predictive model generated herein was validated in an independent set of NSCLC, demonstrating the great potential of our methodology. However, it has limitations. Patients lack information on other comorbidities that may influence body composition and clinical outcomes. Considering that LM patients had a higher frequency of smokers, it is rational to consider that the skeletal muscle alterations identified are triggered by the combination of smoking and CIFs. However, it is difficult to determine the individual impact of each of these variables. We also failed to apply the golden standard definitions of cachexia, such as weight loss, in those patients. Moreover, even though transcriptomic data have high resolution and produce more robust information than proteomic data, the results should be interpreted carefully considering the complex transcriptional regulation in human cells. Therefore, the results of the secretome and ligand-receptor predictions presented in this study need experimental validation in cachectic patients. Furthermore, the immune TME profile of patients with LM patients must be validated by histological examination to check the correlation between CD8 exhaustion and cachexia in a larger number of samples.

## Conclusions

We used systems biology to classify cachexia in treatment-naive NSCLC patients and characterized the TME immune fraction from the LM patients to understand molecular mechanisms by which the tumor induces skeletal muscle atrophy and influences poor outcomes. These results may contribute to the development of therapeutic strategies aiming to decrease muscle atrophy and deleterious inflammation, thus increasing the survival and quality of life of patients with NSCLC cachexia. Overall, the predictive model identified patients with lower muscularity and worse survival, who showed a TME with an inflammatory and immunosuppressive profile, expressing CIFs and potentially enriched with CD8+ T cells. These findings open new perspectives on the early cachexia diagnosis for NSCLC patients. In addition, this study paves the way for the identification of tumor cell-specific cachexia mediators and biomarkers. In the future, we hope that these results can serve as a basis for generating effective treatments for cachexia in patients with lung cancer.

## Supplementary Information

**Additional file 1. **Information regarding software, tools, and databases used in the methodology.

**Additional file 2: Figure S1.** Pectoralis muscle area (PMA) cut-offs predict cachexia. A) Boxplot of PMA (mm^2^) from 38 young males, 97 old males, 34 young females, and 42 old females. B) Boxplot of the same measurements from panel A z-score normalized for gender and age. C) Decision tree generated using Classification And Regression Trees (CART) and Kaplan–Meier survival analyses for down-sampling models. The bottom boxes indicate hazard ratios, the number of patients at risk in each leaf, and the percentage of patients in each leaf. Red-curve: PMA > cut-off and low-risk group; Black-curve: PMA < cut-off and high-risk group. D) Area under the ROC curve (AUC-ROC) that demonstrates the specificity and sensitivity of the PMA skeletal muscle index (PMA cm2/height2) in indicating sarcopenia (determined by the PMA cut-offs generated in Fig. 1A). Male (n = 66) and female (n = 23) groups included both young and older patients that had height information available. **Figure S2.** Exhausted CD8+ T cells interact with pre-exhausted CD8+ T cells through tumor-derived factors identified in low-muscularity patients and negatively correlate with pectoralis muscle areas (PMA). A) Heatmap demonstrating the number of interactions between each CD8 cell subset. CD8_C6-LAYN is the exhausted subtype, while CD8_C4-GZMK corresponds to the pre-exhausted subtype. Analysis was performed using CellPhoneDB using the single-cell RNAseq data publicly available at GSE99254. B) Pearson correlation plot showing a negative correlation between CD8 and exhaustion scores (identified in the digital cytometry analysis) and PMA. BMI: body mass index.

**Additional file 3: Table S1.** Human Protein Atlas (HPA) list of 2943 secretory genes predicted using majority decision-based method for secreted proteins (MDSEC). **Table S2.** Lung cancer cell lines and lung tumor 3D organoids. **Table S3.** List of differentially expressed genes in low-muscularity patients compared to high-muscularity patients. **Table S4.** List of cachexia-inducing factors described in the literature. **Table S5.** List of genes specifically expressed in exhausted tumor CD8+ T cells according to Guo et al., 2018.

## Data Availability

The datasets presented in this study can be found in online public repositories available under the accession numbers GSE103584 and GSE99254. The results shown here are in part based on data generated by the Genotype-Tissue Expression project (GTEx) (https://gtexportal.org/).

## Abbreviations

NSCLC: Non-small cell lung cancer
PMA: Pectoralis muscle area
CIFs: Cachexia-inducing factors
TCIA: The Cancer Imaging Archive
LM: Low muscularity
HM: High muscularity
TME: Tumor microenvironment
CT: Computed tomography
ML: Machine learning
DEGs: Differentially expressed genes
BMI: Body mass index
SMI: Skeletal muscle index
RNA-Seq: RNA sequencing
scRNA-Seq: Single-cell RNA sequencing
