# Supplementary material for: Low muscle mass in lung cancer is associated with an inflammatory and immunosuppressive tumor microenvironment
Source: J Transl Med. 2023 Feb 11;21:116. doi: 10.1186/s12967-023-03901-5 (PMC9921698; doi:10.1186/s12967-023-03901-5)
Supplement: Supplementary file 1 — Additional file 1. Information regarding software, tools, and databases used in the methodology. [file 12967_2023_3901_MOESM1_ESM.pdf]

Additional file. Information regarding the software, tools, and databases used in the methodology.

Software, tools, and databases	Web page	Version	Reference
NSCLC-Radiogenomics collection	https://wiki.cancerimagingarchive.net/display/Public/NSCLC+Radiogenomics		[1]
gcrma human dataset			[2]
Slice-O-Matic	https://www.tomovision.com/products/sliceomatic.html	v.5.0	
Cutoff Finder		v1.0	[3]
rpart	https://cran.r-project.org/web/packages/rpart/index.html	v4.1	
RStudio	https://www.rstudio.com/	v.4.1.0	
R	https://cran.r-project.org/	v3.5.1	
easyROC	http://www.biosoft.hacettepe.edu.tr/easyROC/	v. 1.3.1	[4]
Human Protein Atlas	http://www.proteinatlas.org	v18.1	[5]
Cytoscape	https://cytoscape.org/	v3.7.2	[6]
CytoNCA	https://apps.cytoscape.org/apps/cytonca	v2.1.6	[7]
GTEx portal	https://www.gtexportal.org/	V8	[8]
cellxgene Data Portal	https://cellxgene.cziscience.com	v0.16.0	
The Cancer Imaging Archive	http://cancerimagingarchive.net		[9]
BioGPS	http://biogps.org/		[10]
SankeyMATIC	http://sankeymatic.com/		
Cancer Cell Line Encyclopedia portal	https://portals.broadinstitute.org/ccle		[11]
TCGA-110CL Cell Line Panel	https://comphealth.ucsf.edu/app/tcga-110		[12]
Human Cancer Model Initiative catalog	https://ocg.cancer.gov/programs/hcmi/		
CIBERSORTx	https://cibersortx.stanford.edu/		[13]
Immune Cell Abundance Identifier	http://bioinfo.life.hust.edu.cn/ImmuCellAI#!/		[14]
CellPhoneDB	https://www.cellphonedb.org/		[15]
EnrichR	https://maayanlab.cloud/Enrichr/		[16,17]
Biojupies	https://amp.pharm.mssm.edu/biojupies/		[18]
ClustVis	https://biit.cs.ut.ee/clustvis/		[19]

References

1. Bakr S, Gevaert O, Echegaray S, Ayers K, Zhou M, Shafiq M, et al. Data for NSCLC Radiogenomics Collection [Internet]. The Cancer Imaging Archive; 2017 [cited 2020 Dec 17]. Available from: https://wiki.cancerimagingarchive.net/x/W4G1AQ

2. Su AI, Wiltshire T, Batalov S, Lapp H, Ching KA, Block D, et al. A gene atlas of the mouse and human protein-encoding transcriptomes. Proceedings of the National Academy of Sciences. 2004;101:6062–7.

3. Budczies J, Klauschen F, Sinn BV, Győrffy B, Schmitt WD, Darb-Esfahani S, et al. Cutoff Finder: A Comprehensive and Straightforward Web Application Enabling Rapid Biomarker Cutoff Optimization. van Diest P, editor. PLoS ONE. 2012;7:e51862.

4. Goksuluk D, Korkmaz S, Zararsiz G, Karaagaoglu A Ergun. easyROC: An Interactive Web-tool for ROC Curve Analysis Using R Language Environment. The R Journal. 2016;8:213.

5. Thul PJ, Åkesson L, Wiking M, Mahdessian D, Geladaki A, Ait Blal H, et al. A subcellular map of the human proteome. Science. 2017;356:eaal3321.

6. Shannon P. Cytoscape: A Software Environment for Integrated Models of Biomolecular Interaction Networks. Genome Research. 2003;13:2498–504.

7. Tang Y, Li M, Wang J, Pan Y, Wu F-X. CytoNCA: A cytoscape plugin for centrality analysis and evaluation of protein interaction networks. Biosystems. 2015;127:67–72.

8. Robinson MD, Oshlack A. A scaling normalization method for differential expression analysis of RNA-seq data. Genome Biol. 2010;11:R25.

9. Clark K, Vendt B, Smith K, Freymann J, Kirby J, Koppel P, et al. The Cancer Imaging Archive (TCIA): Maintaining and Operating a Public Information Repository. J Digit Imaging. 2013;26:1045–57.

10. Wu C, Orozco C, Boyer J, Leglise M, Goodale J, Batalov S, et al. BioGPS: an extensible and customizable portal for querying and organizing gene annotation resources. Genome Biol. 2009;10:R130.

11. Barretina J, Caponigro G, Stransky N, Venkatesan K, Margolin AA, Kim S, et al. The Cancer Cell Line Encyclopedia enables predictive modelling of anticancer drug sensitivity. Nature. 2012;483:603–7.

12. Yu K, Chen B, Aran D, Charalel J, Yau C, Wolf DM, et al. Comprehensive transcriptomic analysis of cell lines as models of primary tumors across 22 tumor types. Nat Commun. 2019;10:3574.

13. Chen B, Khodadoust MS, Liu CL, Newman AM, Alizadeh AA. Profiling Tumor Infiltrating Immune Cells with CIBERSORT. In: von Stechow L, editor. Cancer Systems Biology [Internet]. New York, NY: Springer New York; 2018 [cited 2020 Dec 17]. p. 243–59. Available from: http://link.springer.com/10.1007/978-1-4939-7493-1_12

14. Miao Y, Zhang Q, Lei Q, Luo M, Xie G, Wang H, et al. ImmuCellAI: A Unique Method for Comprehensive T-Cell Subsets Abundance Prediction and its Application in Cancer Immunotherapy. Adv Sci. 2020;7:1902880.

15. Efremova M, Vento-Tormo M, Teichmann SA, Vento-Tormo R. CellPhoneDB: inferring cell–cell communication from combined expression of multi-subunit ligand–receptor complexes. Nat Protoc. 2020;15:1484–506.

16. Chen EY, Tan CM, Kou Y, Duan Q, Wang Z, Meirelles G, et al. Enrichr: interactive and collaborative HTML5 gene list enrichment analysis tool. BMC Bioinformatics. 2013;14:128.

17. Kuleshov MV, Jones MR, Rouillard AD, Fernandez NF, Duan Q, Wang Z, et al. Enrichr: a comprehensive gene set enrichment analysis web server 2016 update. Nucleic Acids Res. 2016;44:W90–7.

18. Torre D, Lachmann A, Ma'ayan A. BioJupies: Automated Generation of Interactive Notebooks for RNA-Seq Data Analysis in the Cloud. Cell Systems. 2018;7:556-561.e3.

19. Metsalu T, Vilo J. ClustVis: a web tool for visualizing clustering of multivariate data using Principal Component Analysis and heatmap. Nucleic Acids Res. 2015;43:W566–70.
