# Supplementary material for: Low muscle mass in lung cancer is associated with an inflammatory and immunosuppressive tumor microenvironment
Source: J Transl Med. 2023 Feb 11;21:116. doi: 10.1186/s12967-023-03901-5 (PMC9921698; doi:10.1186/s12967-023-03901-5)

Figure S1. Pectoralis muscle area (PMA) cut-offs predict cachexia. A) Boxplot of PMA (mm^2) from 38 young males, 97 old males, 34 young females, and 42 old females. **B)** Boxplot of the same measurements from panel A z-score normalized for gender and age. **C)** Decision tree generated using Classification And Regression Trees (CART) and Kaplan-Meier survival analyses for down-sampling models. The bottom boxes indicate hazard ratios, the number of patients at risk in each leaf, and the percentage of patients in each leaf. Red-curve: PMA>cut-off and low-risk group; Black-curve: PMA<cut-off and high-risk group. **D)** Area under the ROC curve (AUC-ROC) that demonstrates the specificity and sensitivity of the PMA skeletal muscle index (PMA cm2/height2) in indicating sarcopenia (determined by the PMA cut-offs generated in Figure 1A). Male (n = 66) and female (n = 23) groups included both young and older patients that had height information available.

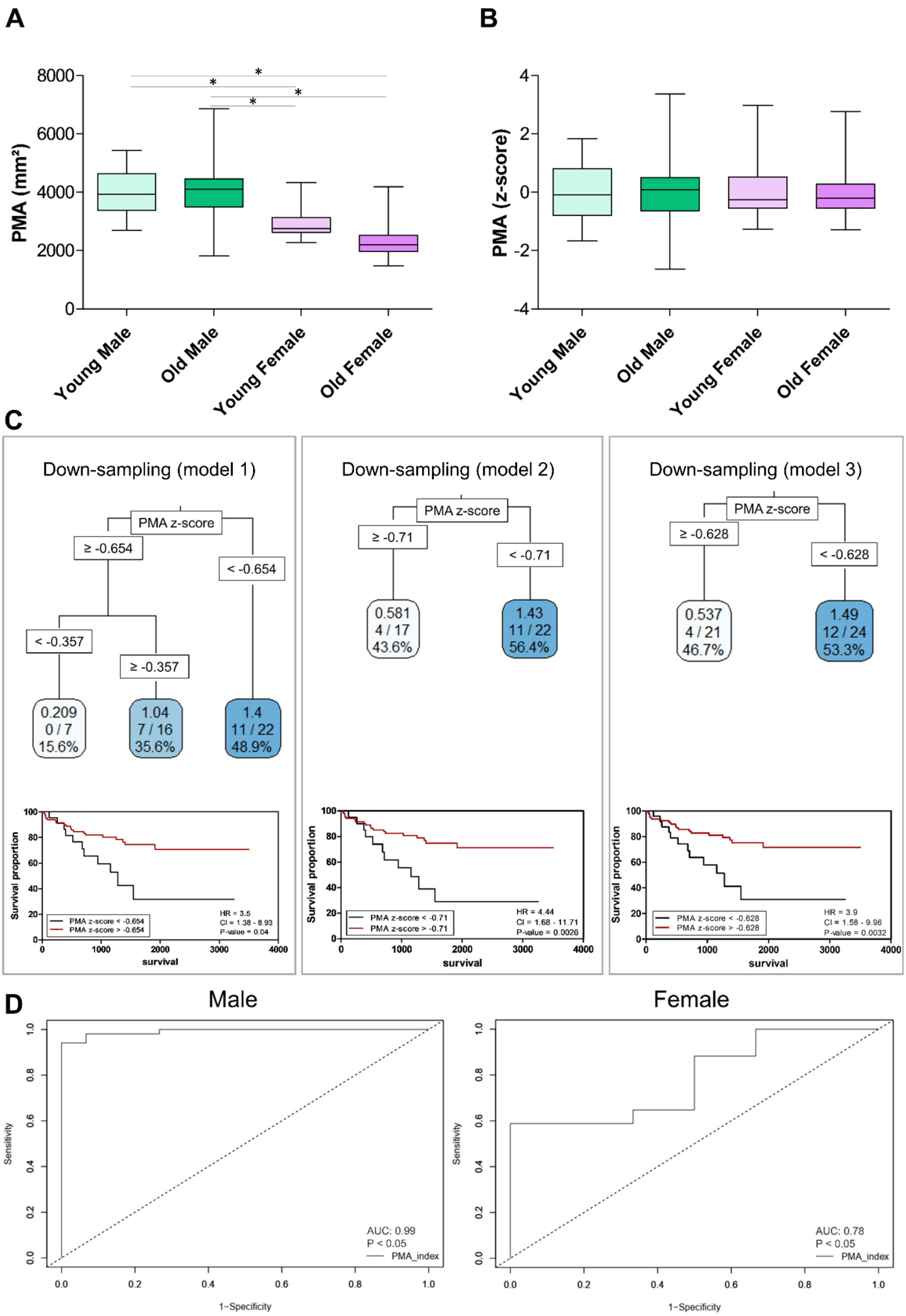
A
PMA (mm²)
8000
6000
4000
2000
0
*
*
*
*
Young Male
Old Male
Young Female
Old Female
B
PMA (z-score)
4
2
0
-2
-4
Young Male
Old Male
Young Female
Old Female
C
Down-sampling (model 1)
PMA z-score
≥ -0.654
< -0.654
< -0.357
≥ -0.357
0.209
0 / 7
15.6%
1.04
7 / 16
35.6%
1.4
11 / 22
48.9%
Survival proportion
survival
PMA z-score < -0.654
PMA z-score > -0.654
HR = 3.5
CI = 1.38 - 8.93
P-value = 0.04
Down-sampling (model 2)
PMA z-score
≥ -0.71
< -0.71
0.581
4 / 17
43.6%
1.43
11 / 22
56.4%
Survival proportion
survival
PMA z-score < -0.71
PMA z-score > -0.71
HR = 4.44
CI = 1.68 - 11.71
P-value = 0.0026
Down-sampling (model 3)
PMA z-score
≥ -0.628
< -0.628
0.537
4 / 21
46.7%
1.49
12 / 24
53.3%
Survival proportion
survival
PMA z-score < -0.628
PMA z-score > -0.628
HR = 3.9
CI = 1.58 - 9.96
P-value = 0.0032
D
Male
Sensitivity
1−Specificity
AUC: 0.99
P < 0.05
PMA_index
Female
Sensitivity
1−Specificity
AUC: 0.78
P < 0.05
PMA_index

Figure S2. Exhausted CD8+T cells interact with pre-exhausted CD8+T cells through tumor-derived factors identified in low-muscularity patients and negatively correlate with pectoralis muscle areas (PMA). A) Heatmap demonstrating the number of interactions between each CD8 cell subset. CD8_C6-LAYN is the exhausted subtype, while CD8_C4-GZMK corresponds to the pre-exhausted subtype. Analysis was performed using CellPhoneDB using the single-cell RNAseq data publicly available at GSE99254. **B)** Pearson correlation plot showing a negative correlation between CD8 and exhaustion scores (identified in the digital cytometry analysis) and PMA. BMI: body mass index.

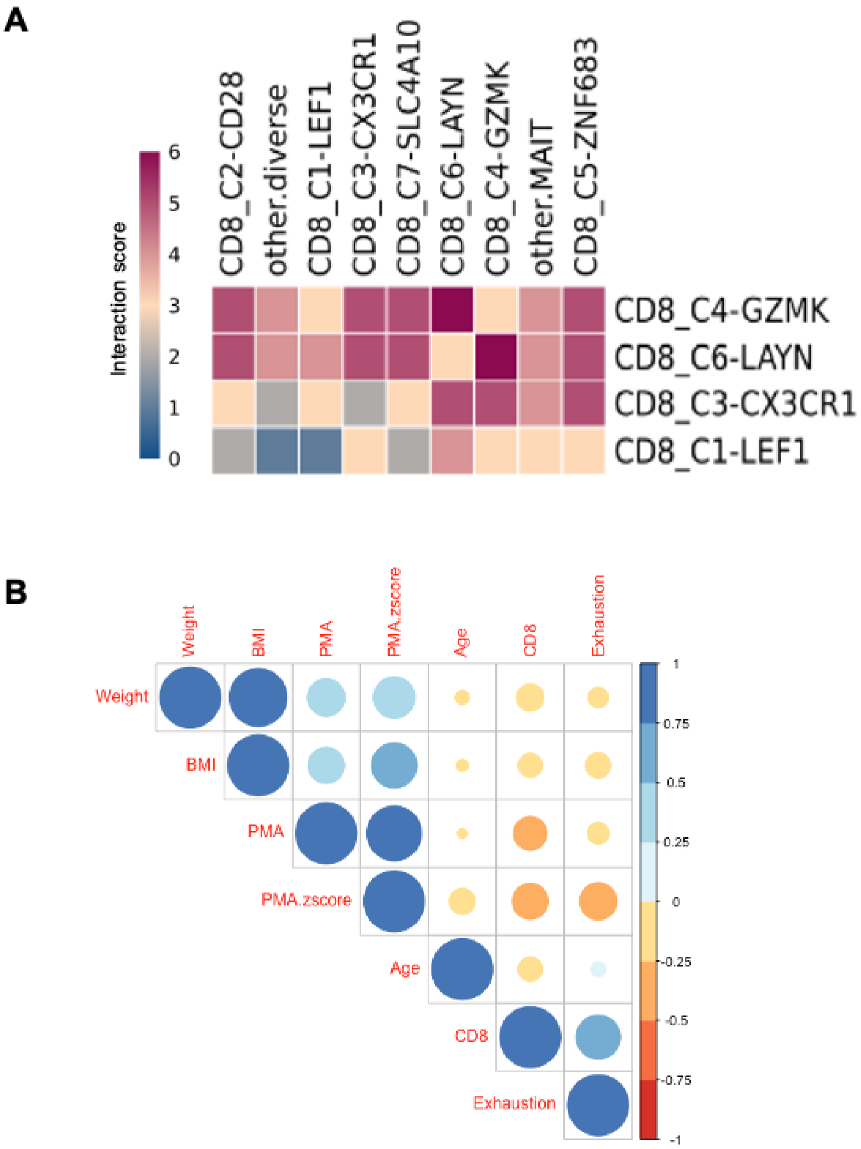

Supplement: Supplementary file 2 — Additional file 2: Figure S1. Pectoralis muscle area (PMA) cut-offs predict cachexia. A) Boxplot of PMA (mm2) from 38 young males, 97 old males, 34 young females, and 42 old females. B) Boxplot of the same measurements from panel A z-score normalized for gender and age. C) Decision tree generated using Classification And Regression Trees (CART) and Kaplan–Meier survival analyses for down-sampling models. The bottom boxes indicate hazard ratios, the number of patients at risk in each leaf, and the percentage of patients in each leaf. Red-curve: PMA > cut-off and low-risk group; Black-curve: PMA < cut-off and high-risk group. D) Area under the ROC curve (AUC-ROC) that demonstrates the specificity and sensitivity of the PMA skeletal muscle index (PMA cm2/height2) in indicating sarcopenia (determined by the PMA cut-offs generated in Fig. 1A). Male (n = 66) and female (n = 23) groups included both young and older patients that had height information available. Figure S2. Exhausted CD8+ T cells interact with pre-exhausted CD8+ T cells through tumor-derived factors identified in low-muscularity patients and negatively correlate with pectoralis muscle areas (PMA). A) Heatmap demonstrating the number of interactions between each CD8 cell subset. CD8_C6-LAYN is the exhausted subtype, while CD8_C4-GZMK corresponds to the pre-exhausted subtype. Analysis was performed using CellPhoneDB using the single-cell RNAseq data publicly available at GSE99254. B) Pearson correlation plot showing a negative correlation between CD8 and exhaustion scores (identified in the digital cytometry analysis) and PMA. BMI: body mass index. [file 12967_2023_3901_MOESM2_ESM.pdf]
